# Characteristics of BAY 2599023 in the Current Treatment Landscape of Hemophilia A Gene Therapy

**DOI:** 10.2174/1566523222666220914105729

**Published:** 2023-01-26

**Authors:** Steven W. Pipe, Valder R. Arruda(Late), Claudia Lange, Stephen Kitchen, Hermann Eichler, Samuel Wadsworth

**Affiliations:** 1 Departments of Pediatrics and Pathology, University of Michigan, Ann Arbor, MI, USA;; 2 Division of Hematology, Department of Pediatrics, Center for Cell and Molecular Therapeutics at Children’s Hospital of Philadelphia, University of Pennsylvania Perelman School of Medicine, Philadelphia, PA, USA;; 3 Bayer AG, Berlin, Germany;; 4 Sheffield Haemophilia and Thrombosis Centre, Sheffield Teaching Hospitals, Sheffield, UK;; 5 Institute of Clinical Hemostaseology and Transfusion Medicine, Saarland University and University Hospital, Homburg/Saar, Germany;; 6 Ultragenyx Pharmaceutical Inc., Novato, CA, USA

**Keywords:** AAV vector, manufacturing, hemophilia A, gene therapy, treatment landscape, factor VIII, BAY 2599023

## Abstract

Hemophilia A, a single gene disorder leading to deficient Factor VIII (FVIII), is a suitable candidate for gene therapy. The aspiration is for single administration of a genetic therapy that would allow the production of endogenous FVIII sufficient to restore hemostasis and other biological processes. This would potentially result in reliable protection from bleeding and its associated physical and emotional impacts. Gene therapy offers the possibility of a clinically relevant improvement in disease phenotype and transformational improvement in quality of life, including an opportunity to engage in physical activities more confidently. Gene therapy products for hemophilia A in advanced clinical development use adeno-associated viral (AAV) vectors and a codon-optimized B-domain deleted FVIII transgene. However, the different AAV-based gene therapies have distinct design features, such as choice of vector capsid, enhancer and promoter regions, FVIII transgene sequence and manufacturing processes. These, in turn, impact patient eligibility, safety and efficacy. Ideally, gene therapy technology for hemophilia A should offer bleed protection, durable FVIII expression, broad eligibility and limited response variability between patients, and long-term safety. However, several limitations and challenges must be overcome. Here, we introduce the characteristics of the BAY 2599023 (AAVhu37.hFVIIIco, DTX 201) gene therapy product, including the low prevalence in the general population of anti-AAV-hu37 antibodies, as well as other gene therapy AAV products and approaches. We will examine how these can potentially meet the challenges of gene therapy, with the ultimate aim of improving the lives of patients with hemophilia A.

## 1 INTRODUCTION

Gene therapy offers the possibility to drive substantial disease modification, greatly improving the quality of life for patients with a number of genetic disorders [1, 2]. Hemophilia A and B, both monogenic coagulation disorders, are suitable targets for gene therapy [3]. Hemophilia is a hemorrhagic disorder characterized by the risk of painful bleeds, typically into joints, soft tissues and even life-threatening locations, which may occur spontaneously, as well as with trauma [4]. Recurrent joint bleeds are associated with complications, including synovitis and arthropathy, leading to long-term joint damage [5, 6]. The disease has a considerable negative impact on the patient’s quality of life [7].

Hemophilia A is more common than hemophilia B (estimated 24.6 cases per 100,000 born males for hemophilia A compared to 5.0 cases per 100,000 males for hemophilia B) [8]. It is characterized by a defective *F8* gene coding for coagulation factor VIII (FVIII), with FVIII levels lower than 1 IU/dL currently defined as severe disease; moderate disease as 1–5 IU/dL, and mild disease as >5–40 IU/dL [4]. Approximately 60% of patients have severe hemophilia A [9]. Human FVIII is an important component of the blood coagulation cascade and has a well-established role in the restoration of hemostasis following tissue injury [10]. In addition, there is an emerging awareness that FVIII may be involved in a range of physiological processes beyond hemostasis, including angiogenesis (potentially in conjunction with von Willebrand factor) and osteoblast development, though its clinical significance needs to be further evaluated [10-13].

Modern hemophilia treatment dates back to the 1970s, when purified, concentrated blood clotting factors started being produced on a commercial scale [14]. In the 1980s, the emergence of hepatitis and human immunodeficiency virus/acquired immune deficiency syndrome (HIV/AIDS) in a large proportion of patients treated with concentrated blood clotting factors led to a need for increased safety measures. The risk of blood infections was reduced further in the 1990s, with an improved understanding of the genetic basis of coagulation disorders leading to the development of laboratory-produced (recombinant) FVIII, generated using DNA technology. The recombinant factors were further refined in the decades that followed, and manufacturing processes were improved to eliminate human and animal proteins in the final products [14, 15].

The standard of care for severe hemophilia A is regular replacement therapy (prophylaxis) with clotting factor concentrates (*e.g*., recombinant FVIII protein) or other hemostasis products to prevent bleeding; on-demand treatment with clotting factor replacement therapy in instances of acute bleeding is available but is not considered to be a long-term treatment option. Standard half-life (SHL) FVIII products have a relatively short half-life and require frequent infusions, representing a significant treatment burden for patients [4]. Further advances during the 2010s led to the development of engineered extended half-life (EHL) FVIII products (increased from approximately 10–14 hours up to approximately 19 hours) [14]. Long-term clinical and real-world evidence has demonstrated that prophylaxis with FVIII replacement products (and EHLs in particular) effectively reduces bleeds, with the aim of allowing patients to live an active life and minimizing the risk of long-term complications [16-18]. In particular, early (childhood) initiation of primary prophylaxis has been associated with fewer joint bleeds and also better future joint health [16, 19, 20].

Nevertheless, a significant unmet need remains. Patients who receive FVIII infusions may develop anti-FVIII antibodies (inhibitors). This occurs more frequently in patients with severe disease and in up to 25–40% of previously untreated patients. The overall inhibitor incidence rate is 2.1 per 1000 person-years for previously treated patients with severe/moderately severe disease who continue to receive recombinant FVIII [4, 21, 22]. Patients with a current inhibitor show a higher mortality rate compared to those without, and may require costly/complex immune tolerance induction therapy and treatment with bypassing agents for eradication, or restoration of hemostasis, respectively [23]. Furthermore, the risk of subclinical bleeds and joint damage remains for all patients with hemophilia, even for patients with no history of overt clinical bleeding [24]. Spontaneous intracranial hemorrhage is a serious risk, especially for patients without access to prophylaxis, and also in children and neonates [25]. Another unmet need is that the use of factor concentrates is associated with the burden of frequent intravenous infusions [26]. Newer, subcutaneously administered, non-factor replacement (NFR) therapy (that mimics the function of FVIII) may provide a suitable option for some patients, particularly those with inhibitors or venous access issues [4]. However, long-term safety and efficacy data are needed, and FVIII replacement is still required (*e.g*., breakthrough bleeds and major surgery) [26]. Currently, while only one NFR is approved for use (emicizumab, Hemlibra^®^), a number of other products are in the development phase [14, 26]. Finally, owing to the significant treatment burden, as well as other barriers to treatment access, low adherence often occurs, which can be a risk factor for worse bleeding outcomes [14, 27, 28].

Gene therapy is the next step in the evolution of hemophilia treatment. It has the potential to address the unmet need in hemophilia A by enabling endogenous production of FVIII and achieving factor levels sufficient to reduce the risk of bleeds and allowing patients to largely stop regular infusions [29, 30]. A number of hemophilia A gene therapy products are expected to reach the market in the next few years [31]. In this review, we describe the gene therapy candidate BAY 2599023 (AAVhu37.hFVIIIco, DTX 201) by summarizing the data-informed design features of the product and how these may address some of the unmet needs in hemophilia A. We also provide a broad comparative overview of other investigational products in hemophilia A gene therapy.

## 2 OVERVIEW OF AAV-DIRECTED GENE THERAPY

Viruses are frequently chosen as gene therapy vectors, owing to their versatility and ability to deliver genetic material mainly to specific target cells (*i.e*., tissue tropism). Early approaches to gene therapy for hemophilia A included both viral and non-viral vectors, with adeno-associated viruses (AAVs) emerging as the main tool for gene therapy for a range of human diseases. Wild-type AAV, a member of the parvovirus family, is composed of a single-stranded linear DNA genome of 4,675 nucleotides (AAV2) encoding replication and structural capsid genes, flanked by inverted terminal repeat (ITR) sequences [32]. This is contained in a non-enveloped capsid comprised of three different cap proteins arranged into a 60-mer icosahedron [33]. AAV serotypes with structural and serologically similar characteristics are grouped and classified by clade (A–F) [34]. However, the total number of serotypes, either naturally occurring (*e.g*., AAV2) or synthesized (designated with a hyphen, such as AAV-NP59), is unknown, with over 100 serotypes identified [34]. In recombinant AAV-based gene therapy, a bioengineered expression vector containing the target gene expression cassette and viral ITRs (in place of the viral protein-coding sequences) is pseudo-packaged into the chosen AAV serotype capsid [35].

AAVs are thought to be non-pathogenic, lacking the essential genes required for replication and expression of the genome, and relying instead on the co-infection of a helper virus for active infection [36]. AAV vectors have several other characteristics that are beneficial for their use in gene therapy, including tropism for a range of cell types, and the ability to transduce both actively proliferating cells as well as those no longer undergoing division [37, 38]. Wang *et al.* have comprehensively reviewed the history of the development of recombinant AAV vectors for gene therapy, from the first cloning of the wild-type AAV sequence into plasmids to the first approved AAV gene therapies in humans [39].

Successes with AAV-based gene therapies include the first US Food and Drug Administration (FDA)-approved AAV, for Leber congenital amaurosis type 2 due to *RPE65*-mediated inherited retinal degenerative disease (voretigene neparvovec, Luxturna^®^). Voretigene neparvovec contains an AAV2 vector with a functional copy of the *RPE65* gene. Another AAV-based product was approved in 2019 for the treatment of spinal muscular atrophy type 1 (onasemnogene abeparvovec, Zolgensma^®^). This gene therapy used an AAV9 vector with a transgene encoding the human survival motor neuron protein. Both products contained a cytomegalovirus enhancer and chicken β-actin hybrid promoter [40]. These products have been shown to provide durable efficacy over several years, with a favorable safety profile [41, 42], thus providing compelling evidence for using an AAV-based gene therapy approach.

## 3 AAV LIVER-DIRECTED GENE THERAPY IN HEMOPHILIA A

### 3.1 Background

The first wave of licensed gene therapies for hemophilia A will likely use an AAV-based liver-directed approach. Although FVIII is mainly produced and then secreted from liver sinusoidal endothelial cells [43, 44], the FVIII transgene is delivered to hepatocytes. Hepatocytes have shown promising expression and secretion in preclinical studies despite not being the natural site of FVIII expression. Following a single outpatient intravenous infusion over as little as 1–3 hours, these particles are internalized *via* receptor-mediated endocytosis; the capsid undergoes a conformational change, and the genome is shuttled to the nucleus, forming a concatemerized circular episome that confers stability to the AAV genome [45]. In the nucleus, genetic elements that accompany the gene allow for efficient expression and ultimate secretion of FVIII protein into the plasma. Ultimately, a steady state between secretion and clearance represented by a factor activity level is reached and can be measured with traditional blood sampling.

Hemophilia A AAV-based gene therapy products in development are a unique combination of intrinsic proprietary design features, including capsid serotypes, transgene engineering, promoters and enhancers, as well as manufacturing platforms, optimized for safety and long-lasting efficacy. Considerable efforts have been devoted to optimizing these features, as well as other variables, such as vector dose.

### 3.2 AAV Capsid Serotype Selection

The selection of an appropriate AAV for gene therapy is largely based on the required and desired properties conferred by a specific capsid serotype. Serotype-specific differences have been shown to affect immunogenicity, transduction efficiency, transgene expression and tissue tropism [35, 37, 46-49]. AAV tissue tropism is partially dictated by an interaction between the capsid and receptors expressed on target cells, including proteoglycans [50, 51], with non-receptor-mediated cell entry also possible [52]. AAV tissue tropism is difficult to assess in humans, given the need for biopsies or labelled capsids for imaging, but data from animal models may provide an insight. However, AAV tissue tropism varies in different animal models [53], so it is unclear whether the profiles will correlate with human tropism. Humanized liver animal models [49, 54] offer an additional alternative means to test eventual *in vivo* human hepatocyte tropism of AAV.

### 3.3 Genetic Engineering of the FVIII Transgene

Bioengineering approaches to develop the optimal expression cassette play a pivotal role in the success of gene therapy. Both codon optimization at the DNA level or manipulations to affect the resultant protein are possible. A number of hemophilia B gene therapy products are at a more advanced stage of clinical development than for hemophilia A largely due to the lower complexity and size of the Factor IX (FIX) gene compared to that for FVIII; developing gene therapy techniques for the more prevalent hemophilia A form of the disease has been a considerable challenge [55-57]. Since the FVIII gene was first characterized in 1984 [56, 58], many years of dedicated research have underpinned the bioengineering of the FVIII transgene. In 2006, Jiang and colleagues demonstrated that FVIII was expressed at physiologic levels in a murine hemophilia A model using AAV serotypes 6 and 8 [59]. In addition, FVIII was persistently expressed with AAV serotypes 2, 6 and 8 in hemophilia A canine model, with no toxicity observed [59].

Initial challenges in packaging and delivering hemophilia A gene therapy focused on the large FVIII transgene size (coding region of approximately 7Kb) [58, 60]. However, the packaging capacity of AAV vectors is approximately 4.7 Kb [61]. Naturally expressed FVIII is a large, complex glycoprotein with six structural domains, including the B domain [62]. Deletions of the B domain have shown to have no effect on the coagulation activity of FVIII, neither *in vitro* nor *in vivo*; therefore, the use of B-domain truncated or B-domain deleted FVIII products minimized previous sizing constraints [63, 64]. The deleted B-domain may also be replaced with synthetic linkers, such as SQ and V3, which enhances AAV-derived FVIII expression [65-67]. Furthermore, codon optimization is important, particularly if targeted at a specific cell type. For example, expression of a liver codon-optimized FVIII construct (An53) was two-fold higher in cell culture than for standard human codon optimization [68].

### 3.4 Promoters and Enhancers

In hemophilia A gene therapy, liver-specific promoter sequences derived from hepatocyte-synthesized proteins have been used to limit expression to one tissue type and may be additionally optimized to increase specificity. Examples of liver-specific promoters include transthyretin (TTR) [69] and albumin [70], as well as the apolipoprotein E (enhancer)/human alpha-1 antitrypsin (hAAT; promoter) combination [71]. Synthetic liver-specific promoters have also been engineered, such as the 146 bp hepatic combinatorial bundle (HCB) [68]. It should be stressed that preclinical studies informing clinical trials for hemophilia A AAV-based gene therapy used hepatocyte-specific (*i.e*., not all types of liver cell) promoters [43, 44].

### 3.5 Manufacturing

Several biological production platforms have been used for hemophilia A gene therapy, including both mammalian and non-mammalian systems. These have seen recent advances in terms of both large-scale production of AAV vectors and also improvements in quality control. Optimal manufacturing and validation processes are critical, including ensuring the minimization of impurities, such as residual host cell proteins and empty capsids [72].

Mammalian systems include human embryonic kidney (HEK)293 and HeLa cell lines [73, 74]. In preclinical studies, adherent HEK293 cells are typically co-transfected with two or three plasmids, encoding the vector genome, *rep* and *cap* genes, and adenoviral helper genes. This method is challenging for large-scale production of AAV but can be overcome by using AAV packaging and producer cell lines adapted for growth in suspension [74, 75]. The latter contain *rep* and *cap* genes, as well as the vector sequence, and only require infection with helper virus. A manufacturing platform using baculovirus-based *Spodoptera frugiperda* Sf9 insect cells has also been developed [76]. Despite also growing in suspension, the resultant AAV vectors have less potency compared to mammalian systems [49]. Furthermore, research has emerged demonstrating differences in post-translational modifications (acetylation, methylation, phosphorylation, O-GlcNAcylation) between the mammalian and insect platforms, which can impact protein stability, targeting, activity, and immunogenicity [49]. Given the differences observed between platforms, ongoing research into how production variables influence vector lot composition, efficacy and safety, is needed.

## 4 LIMITATIONS AND CHALLENGES IN HEMOPHILIA A GENE THERAPY

### 4.1 Restricted Patient Eligibility

#### 4.1.1 Pre-existing Immunity

Pre-existing immunity to AAV is observed in a proportion of the human population as a result of previous exposure to natural AAV infections. Different AAV serotypes have varying levels of pre-existing anti-AAV antibodies, and many studies have investigated population seroprevalence in various settings. These have used different assays and approaches for the assessment of neutralizing and/or binding anti-AAV antibodies. A detailed description of all the published data is outside the scope of this review, but relevant seroprevalence studies are discussed later.

Neutralizing antibodies can mediate AAV capsid neutralization *via* numerous mechanisms, including blocking cell surface attachment and uptake or inhibition of capsid uncoating [77, 78]. Non-neutralizing anti-AAV antibodies also exist, which do not block the uptake of AAV vectors. Their role is not well-defined, but it is thought that they increase capsid clearance processes *via* activation of macrophages and opsonization processes [77, 79]. Pre-existing anti-AAV antibodies in patients with hemophilia A have major implications for gene therapy, as they prevent broad patient eligibility (*i.e*., a high seroprevalence for a given AAV serotype will limit the number of patients who may benefit from gene therapy using that AAV serotype). If pre-existing immunity against the chosen AAV capsid is of concern in terms of efficacy and safety, the contemporaneous development of a companion diagnostic detecting anti-AAV antibodies is required to support the use of the corresponding therapeutic product [80].

#### 4.1.2 Excluded Populations

Hemophilia A gene therapy trials to date have been limited to specific patient populations. For example, they generally exclude children, as well as patients with detectable anti-AAV/neutralizing antibodies, FVIII inhibitors, active hepatitis B virus (HBV), active hepatitis C virus (HCV), active HIV infection, or other hepatic co-morbidities [81]. Therefore, if AAV-based gene therapy proves to be effective and well tolerated in the clinical trial population, further studies will be needed to investigate whether it will also be of benefit to a broader group of patients with hemophilia A.

### 4.2 Re-treatment

The ideal gene therapy only needs to be administered once. However, as the genome mainly exists as a non-integrated episome [82, 83], dilution of the episome and a decrease in transgene expression will occur in replicating hepatocytes [48, 83]. This phenomenon is of particular importance in children where hepatocyte proliferation is most prominent, but it also relates to adults as hepatocytes can continue to divide throughout life [84]. Episomal dilution is of major clinical relevance, as re-dosing may be required to replenish episomes, and thus, FVIII levels. However, this is problematic due to the development of anti-AAV neutralizing antibodies following the initial administration. As the capsid is the gene transfer vehicle, it has direct contact with the host environment and elicits an immune response [85]. Indeed, AAV-mediated gene transfer usually generates the release of high-titer neutralizing antibodies against the viral capsid [86]. These can cross-react with other serotypes [86], so it may not be possible to re-dose with an AAV-based gene therapy using a different serotype.

Possible solutions for this limitation are being tested, including research into nanoparticles containing the immunomodulator rapamycin [87] and AAV anti-immunoglobulin enzymes, such as IdeS (Immunoglobulin G-degrading enzyme of *Streptococcus pyogenes*) [88, 89] and IdeZ (immunoglobulin-degrading *enzyme* from *Streptococcus equi* subspecies *zooepidemicus*) [90]. A further approach is AAV-specific plasmapheresis to selectively reduce AAV-specific antibodies [91].

### 4.3 Durability of Efficacy

The other significant clinical implication of dilution of AAV episomes, and thus loss of transgene expression, relates to the durability of FVIII production. A key factor unknown is how long following administration of AAV-based gene therapy, episomes will remain at a high enough copy number to generate therapeutic levels of FVIII. In this regard, it is notable that AAV-based FVIII transgene expression has been shown for up to 10 years in a canine hemophilia A model [92]. In the clinical setting, the potential of AAV-based gene therapy to produce a durable response is exemplified by the treatment of severe hemophilia B. In this case, stable therapeutic expression of FIX using a serotype 8 AAV (scAAV2/8-LP1-hFIXco) has been shown over 8 years. This was accompanied by a 66% and 82% decrease in annual FIX usage and annualized bleeding rate (ABR), respectively, compared to pre-treatment [93]. Data on the durability of AAV-based gene therapy for hemophilia A in clinical trials to date are discussed later.

Other areas of uncertainty include the extent to which the durability of FVIII expression differs between patients, and the optimal endpoints to assess the long-term response. As well as FVIII levels and ABR, it may be more relevant to assess other endpoints, such as the need for FVIII infusions following gene therapy.

### 4.4 Variability of Response

Interindividual variability in the pharmacokinetics of FVIII replacement therapy products exists; for example, in half-life, which is dependent on factors such as age, blood group and level of von Willebrand factor antigen [94]. It has been hypothesized that hemophilia is suitable for gene therapy, as bleeding responds to a wide range of factor levels, with precise regulation not needed [3]. Variations in the FVIII levels expressed by different patients following the same dose of gene therapy are also expected. Although studies are required, it may be that this variation will be dependent on lifestyle, genetic and environmental factors, as well as age. Despite this lack of data, it can be hypothesized that one type of hemophilia A gene therapy or dose is unlikely to be suitable for all patients, and personalized therapy will be at the center of this approach.

### 4.5 Safety

The safety aspects of administering high doses of AAV vectors to humans require careful consideration and monitoring.

#### 4.5.1 Short-term

The fact that AAV capsids can induce neutralizing antibodies has already been mentioned, but the immune response is much more complex. Innate immunity is critically important, including the interaction of the single-stranded DNA genome of AAV with Toll-like receptor (TLR)9-MyD88, leading to the production of type I interferon [95]. This pathway is crucial for the development of CD8^+^ T-cell responses to both the capsid and transgene product, and loss of transgene expression [95]. Cytosine-guanine oligodeoxynucleotides (CpGs), which are mainly methylated and at low frequency in mammalian cells, are pathogen-associated molecular patterns (PAMPs) that act *via* TRL9 to induce an innate immune response [96, 97]. In a hemophilia B gene therapy trial, it was suggested that the introduction of CpG clusters following optimization of the FIX coding sequence stimulated the innate immune response, and thus loss of transgene expression [97]. This reinforces the need for careful evaluation and testing of codon optimization of therapeutic transgenes. An additional consideration is that supraphysiological AAV-derived FVIII expression resulted in cellular stress in mice, and may require further research [98].

A key short-term safety concern is transient transaminitis, though a sustained effect is also a possibility. In a Phase 1/2 clinical trial in 7 adult patients with severe hemophilia B, 2 patients developed elevated aspartate aminotransferase (AST) and alanine aminotransferase (ALT) levels, beginning 4 weeks after infusion of an AAV2 vector expressing FIX transgene [99]. This may have been due to the destruction of transduced hepatocytes resulting from cell-mediated immunity targeting the AAV capsid. Transaminitis can be treated with corticosteroids, and is covered in more detail in a later section describing clinical trials for AAV-based gene therapy for hemophilia A.

Of relevance, in a Phase 1/2 trial for X-linked myotubular myopathy (NCT03199469) [99], 3 deaths occurred in pediatric patients with pre-existing liver disease following intravenous infusion of AAV-based gene therapy. This product (AT132) used a functional copy of the *MTM1* gene introduced into skeletal muscle cells to produce the missing myotubularin protein under a muscle-specific desmin promoter [100]. Two deaths were reportedly due to sepsis, while one was due to a gastrointestinal bleed, all of which resulted from liver failure [101, 102]. More recently, the fourth death in this same trial occurred, but the cause has been unknown at the time of this writing. Despite concerns about liver damage, preclinical studies of distinct AAV serotypes in hemophilia A murine models have demonstrated no evidence of liver dysfunction following the expression of FVIII [98, 103].

#### 4.5.2 Long-term

Recombinant AAV vectors have a low risk of genomic integration [83, 104]. However, in 2007, a preclinical AAV-based study using mice reported hepatocellular carcinoma (HCC) development [105]. Furthermore, HCC was recently detected in one patient following treatment with the AAV5-based gene therapy, etranacogene dezaparvovec, for hemophilia B in the HOPE-B Phase 3 trial (NCT03569891). Patients with a history of, or well-controlled, hepatitis B or C infection were permitted in HOPE-B. This patient population had several HCC risk factors, and extensive genomic analysis concluded that the HCC was unlikely related to the AAV vector [106]. It is reassuring that no evidence of tumors or altered liver function was found following AAV-based gene therapy in a long-term canine hepatitis A model [92]. Nevertheless, the theoretical possibility of AAV gene therapy-related cancer needs to be closely monitored in clinical trials. Indeed, ultrasound analysis of the liver will be increased from once to twice yearly in HOPE-B to monitor the HCC risk following treatment with etranacogene dezaparvovec [106].

### 4.6 Assessing FVIII Activity Following Gene Therapy

Assessing the benefits of the different gene therapy approaches for hemophilia A in terms of initial FVIII activity levels achieved, the durability of response is not straightforward, as the type of reagent and assay methodology may influence assay results. In hemophilia A, both one-stage activated partial thromboplastin time (aPTT; time to clot formation) and two-stage chromogenic assays are used to determine FVIII activity levels [107, 108]. However, recent studies have reported discrepancies in transgene-FVIII activity between the two methods [109]. One-stage assay results are usually higher than for chromogenic assays, and this discrepancy has been explained by an accelerated FXa and Factor IIa formation causing a kinetic bias between the two assays [110].

In the study by Rosen *et al.*, the specific activities (measured by enzyme-linked immunosorbent assay) of recombinant-derived and transgene-produced FVIII were comparable when assessed by the chromogenic assay, but not the one-stage assay [110]. The authors highlight the vulnerability of one-stage assays for monitoring non-native FVIII, with their results supporting the broad use of chromogenic assays in this setting [110]. However, it is advisable to include results of one-stage as well as chromogenic assays when reporting patient factor activity, and to include full details of the reagents used.

## 5 CHARACTERISTICS OF THE BAY 2599023 (AAVHU37.HFVIIICO) GENE THERAPY PRODUCT

### 5.1 Product Features

#### 5.1.1 AAV Capsid Serotype Selection

BAY 2599023 (AAVhu37.hFVIIIco, also known as DTX 201, Bayer/Ultragenyx) is a novel AAV-based gene therapy for the treatment of severe hemophilia A. Its carefully selected design features aim to address the unmet needs in hemophilia A gene therapy discussed in this review. BAY 2599023 features reflect the recent advancements in AAV technology and hope to overcome obstacles identified in earlier hemophilia A gene therapy studies. It remains to be seen whether these features will translate into clinical benefits.

Based on the results of preclinical models, BAY 2599023 has been designed with the goal of durable expression of clinically meaningful levels of FVIII, leading to avoidance of low trough levels and prevention of bleeding [111, 112]. BAY 2599023 is the first gene therapy for hemophilia A to include the Clade E AAV-hu37 capsid serotype [113].

In FVIII knock-out mice studies, FVIII activity was assessed using the E06.TTR.hFVIIIco-SQ construct in the following vector capsids: AAV8, AAV9, AAVrh10, AAV-hu37, and AAV-rh64R1 [112]. FVIII activity ranged from 0.51 IU/mL for AAV-rh64R1 to 1.26 IU/mL for AAVrh10 at 2 weeks following vector administration [112]. AAVrh10, AAV-hu37, and AAV-rh64R1 generated anti-FVIII antibodies in greater than 20% of mice, with AAV-hu37 at an intermediate level. AAVrh10 was chosen for further evaluation, given that it generated the highest FVIII activity, as well as AAV-hu37 as it had intermediate FVIII expression and immunogenicity [112]. In cynomolgus macaques, the AAV-hu37 capsid generated substantially greater FVIII expression than AAVrh10 for the FVIII transgenes tested [111]. Overall, AAV-hu37 was the least immunogenic capsid tested in both mice and non-human primates.

#### 5.1.2 Seroprevalence Data

As discussed previously, low seroprevalence for an AAV serotype is desired to limit the immune response against the capsid and ensure that many patients can benefit from the gene therapy treatment. In 2010, Wang *et al.* showed a seroprevalence rate for AAV-hu37 neutralizing antibodies of approximately 15% (based on a titer of greater than 1:20) in 40–100 human serum samples [114]. More recently, the seroprevalence of AAV-hu37 was assessed by determining neutralizing antibody levels according to cellular transduction inhibition in 100 US patients with hemophilia A. Neutralizing antibodies against AAV5 and AAV8 were also measured [115]. The study showed that AAV-hu37 had a low pre-existing neutralizing antibody prevalence. Based on a neutralizing antibody titer of lower than 1:5, 86% of patients in this cohort would be eligible for AAV-hu37-based treatment, suggesting broad patient eligibility for this gene therapy product [115]. In contrast, 74% and 60% of patients in this cohort would be able to be treated with an AAV5- or AAV8-based gene therapy, respectively [115].

#### 5.1.3 Transgenes, Promoters and Enhancers

BAY 2599023 comprises a codon-optimized, human FVIII transgene with the B domain replaced with a 14-amino acid SQ linker (BDD-hFVIIIco-SQ) [111]. The preclinical studies, which were conducted to select the capsid, also identified the optimal liver-specific enhancer and promoter combination [111, 112]. Forty-two enhancer/promoter combinations were tested, with 14 enhancers inserted upstream of three promoter regions: TBG-S1 (shortened liver-specific thyroxine-binding globulin), A1AT (modified SERINA1 [alpha-1-antitrypsin]) or TTR [111, 112]. When tested in knockout mice, the highest FVIII activity levels were observed in genome constructs E03.TTR, E05.A1AT, E05.TTR, E06.TTR, and E12.A1AT. Constructs E03.TTR and E12.A1AT were chosen for further evaluation in cynomolgus macaques due to their smaller genomes coupled with higher observed FVIII expression, compared to the others investigated. The constructs were administered with either AAV-hu10 or AAV-hu37 vectors. The AAV-hu37.E03TTR combination emerged as the one with peak FVIII expression levels (20.2 ± 5.0% of normal), with anti-FVIII antibodies not detected in two out of five animals at 30 weeks following vector administration [111]. This combination is used in BAY 2599023.

#### 5.1.4 Manufacturing

AAV-hu37 is suitable for scalable purification in manufacturing, as it has a high affinity for chromatography resin [116]. In addition, it was also shown to produce significantly higher yields than AAV-rh10 (p<0.05) [111]. A proprietary HeLa PCL platform is used to manufacture BAY 2599023, which is considered optimal for large-scale AAV manufacture.

### 5.2 Phase 1/2 Clinical Trial

A single infusion of the BAY 2599023 gene therapy product is currently being investigated in a multinational, open-label, Phase 1/2 dose-finding clinical trial (NCT 03588299) [113, 117]. Early data from a small number of patients are available, but the trial is ongoing. Enrolled patients (up to 30) are previously treated (>150 exposure days to FVIII products) adult (≥18 years old) males with severe hemophilia A and no detectable neutralizing antibodies to AAV-hu37 [117]. The primary endpoint of the clinical trial is the occurrence of adverse events (AEs) and serious AEs (SAEs) up to 52 weeks, including treatment-emergent AEs, and AEs or SAEs of special interest; the secondary endpoint is a change in FVIII activity from baseline up to 5 years and the proportion of patients who reached an expression of FVIII greater than 5% at 6 and 12 months from baseline [113, 117]. Preliminary data available from 8 of 9 patients (cut-off May 2021) were reported for three cohorts (0.5x10^13^ genetic copies [GC]/kg; 1x10^13^ GC/kg; and 2x10^13^ GC/kg, with or without prophylactic steroids) [118]. Sustained FVIII expression was delivered (up to >23 months), with evidence of bleed protection. Elevations in ALT were observed. A fourth cohort who will receive a higher dose of BAY 2599023 (4x10^13^ GC/kg) has now been added to the study design. It is hoped that the selected design features and manufacturing processes will translate to the intended clinical benefits in the anticipated clinical trial data.

## 6 OTHER APPROACHES TO INVESTIGATIONAL HEMOPHILIA GENE THERAPY PRODUCTS

Although AAV transgene delivery remains the main technology currently under investigation for hemophilia A, other approaches, such as lentiviral vectors and gene editing, are also being examined.

### 6.1 AAV Vectors Other than BAY 2599023

**Fig. (1):**
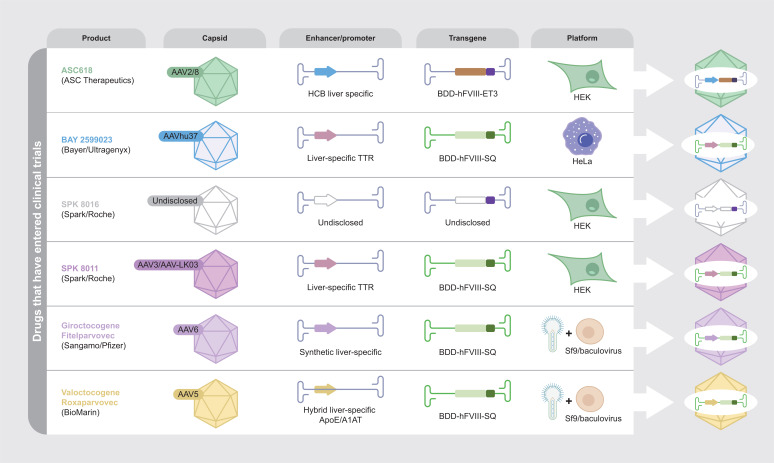
Comparison of hemophilia A, AAV-based gene therapy products in clinical trials. AAV, adeno-associated virus; BDD, B-domain-deleted; HCB, hepatic combinatorial bundle; HEK, human embryonic kidney; hFVIII, human Factor VIII; Sf, *Spodoptera frugiperda*; TTR, transthyretin.

In addition to BAY 2599023, several other AAV-based gene therapies are in development across clinical trial Phases 1 to 3 (Fig. **1**). Hemophilia A AAV-based gene therapy products for which data were available at the time of publication also contain a B-domain-deleted FVIII transgene and are targeted to the liver (where disclosed). As discussed, the capsid, enhancer/promoter, transgene and manufacturing platform differ between the various products. The extent to which these differences will translate into overcoming challenges associated with gene therapy for hemophilia A remains to be elucidated.

Valoctocogene roxaparvovec (Roctavian^®^, formerly Valrox^®^ and BMN-270; Biomarin Pharmaceutical) and giroctocogene fitelparvovec (SB-525 or PF-07055480; Sangamo/Pfizer) are being investigated in Phase 3 clinical trials. Valoctocogene roxaparvovec is a codon-optimized AAV5-based gene therapy that uses the Sf9 baculovirus manufacturing platform [109]. The SQ variant of B-domain-deleted FVIII (FVIII-SQ) is expressed using a hybrid liver-specific promoter [109]. Following infusion of valoctocogene roxaparvovec in cynomolgus monkeys, pre-existing anti-AAV5 antibodies were associated with a reduction in FVIII-SQ maximal concentration and area under the curve compared to non-immune control animals [119]. In a seroprevalence study, anti-AAV5 neutralizing antibodies were detected in 54.4% of patients with severe hemophilia A (N=194) or moderate/severe hemophilia B (N=48) [120]. Further insight will be provided by the observational seroprevalence of AAV antibody (SAAVY) study (NCT04560933), which aims to characterize seroprevalence of AAV5, AAV6 and AAV8 antibodies, and seroconversion rates over time in patients with hemophilia A [113].

A Phase 1/2 ongoing trial (NCT02576795) enrolled 13 adult patients with severe hemophilia A without detectable pre-existing anti-AAV5 antibodies who received a single infusion of valoctocogene roxaparvovec (4 or 6 x10^13^ vg/kg) [121]. FVIII activity levels peaked at year 1 and declined in year 2, followed by a consistent and more predictable decline up to 5 years post-infusion. For example, mean FVIII activity at week 52 for the 6 x10^13^ vg/kg dose was 64.3 IU/dL by chromogenic substrate assay, which decreased to 36.4 IU/dL at week 104 and 11.6 IU/dL at week 260 [121] (above the level defining mild disease [<5-40 IU/dL]) [4]. The mean ABR was reduced by 92%-95% at year 4/5 compared to baseline [121]. AEs were commonly transient, asymptomatic and ALT elevations. All patients remained free of prophylaxis at the 4/5-year follow-up. The Phase 3, single-arm, open-label GENEr8-1 trial (NCT03370913) involved 134 adult patients with severe hemophilia and no detectable pre-existing AAV5 antibodies treated with valoctocogene roxaparvovec at a dose of 6 x10^13^ vg/kg [122]. FVIII activity increased by a mean of 41.9 IU/dL at weeks 49-52 (p<0.001), compared to baseline. In addition, mean ABR decreased by 84% and FVIII infusion rates by 99% after week 4 [122]. A total of 115/134 (86%) patients experienced ALT elevations; 95.6% resolved following treatment with corticosteroids and/or other immunosuppressants. Initial US Food and Drug Administration (FDA) approval for valoctocogene roxaparvovec was rejected in August 2020, with 2 years of Phase 3 trial data required to provide substantial evidence of durable efficacy using ABR as the primary endpoint [123]. An additional post-hoc analysis with a follow-up of 71.6 weeks was performed; at weeks 49–52, 71% (95/134) had median FVIII activity ≥15 IU/dL, and 75% of patients were bleed-free up to the data cut [124].

Giroctocogene fitelparvovec is an AAV6-based gene therapy also manufactured using the Sf9 baculovirus system [125, 126]. The Phase 1/2 ALTA dose-ranging study (NCT03061201) is investigating four doses in adult patients with severe hemophilia and no neutralizing antibodies (presumably against AAV6): 9x10^11^ vector genomes (vg)/kg, 2x10^12^ vg/kg, 1 x10^13^ vg/kg, and 3x10^13^ vg/kg [113]. Interim results (up to 195 weeks in 11 patients, with a cut-off date of May 2021) showed dose-dependent, sustained FVIII expression with ABR of 0 in the first year and 0.9 throughout the entire follow-up period. A total of 3 bleeding events were reported in 2 patients in the highest dose cohort and required exogenous FVIII treatment. No FVIII inhibitors or thrombotic events were reported. AEs included elevated liver enzymes and infusion-related reactions, notably increased ALT (5/11, 45.5%). Treatment-related SAEs were reported in 1 patient in the highest dose cohort, who experienced hypotension and fever, and which resolved with treatment. The results of the pivotal 5-year, Phase 3 AFFINE trial (NCT04370054) in patients with moderately severe or severe hemophilia A that started in August 2020, are eagerly anticipated [113]. However, there have been concerns that some patients receiving giroctocogene fitelparvovec had FVIII levels of more than 150% of normal, which may increase thrombotic risk (though no events have been reported to date) [127]. The trial has been put on clinical hold by the FDA until a protocol amendment is implemented and approved by the health authorities [127].

As well as BAY 2599023, two other gene therapy products are currently in Phase 1/2 trials [113]: SPK 8016 and SPK 8011 (Spark Therapeutics) [128, 129]. In addition, a Phase 1/2 study is planned for ASC-618 (ASC Therapeutics) [130]. The latter uses an HCB-ET3-LCO construct that contains human B-domain deleted FVIII with porcine A1 and A3 sequences that demonstrated a 10-to-100-fold increase in FVIII biosynthesis compared to other FVIII constructs.

Focusing on the Phase 1/2 dose-escalation SPK 8011 clinical trial, with four dose cohorts ranging from 5x10^11^ vg/kg to 2x10^12^ vg/kg, 16 adult patients with hemophilia A showed sustained FVIII expression, with 12 patients followed-up for over 2 years [128]. However, a further 2 patients lost FVIII expression owing to an anti-AAV cellular immune response, which led to a change in protocol for the remaining five patients to include prophylactic glucocorticoids. Analysis involving all 18 patients showed that ABR was reduced by 91.5% and the annualized number of FVIII infusions by 96.4%, compared to pre-treatment. Transient elevated ALT levels treated with glucocorticoids were observed in 7 patients; all were mild, apart from 1 patient in whom this was classified as an SAE. Major safety concerns were not reported [128]. The authors contrasted the generally stable FVIII expression observed with SPK 8011 to the decline over 4/5 years reported with valoctocogene roxaparvovec [121]. They speculated that expression from heterogeneous cell types may contribute to differences in the durability of FVIII expression [128]. In addition, higher FVIII levels following treatment could potentially induce an unfolded protein response leading to a loss of expression [128] (Table **1**).

### 6.2 Product Comparison Limitations

Comparisons of different AAV gene therapy products need to be interpreted with caution, as patient characteristics can differ across studies. Seroprevalence data should not be generalized across different patient populations, where, for *e.g*., country-specific trends can be observed [131]. Seroprevalence assay methods are also not standardized across clinical studies [77, 131]. Finally, clinical trial sample sizes are still small, and further clinical data are needed to confirm whether these varied design approaches meet patient needs within gene therapy with a favorable risk:benefit profile.

### 6.3 Lentiviral Vectors

Approaches beyond AAV-based gene therapy, such as recombinant HIV-derived lentiviral vectors, are also under investigation (recently reviewed by Cantore and Naldini) [132]. Lentiviruses have the potential to overcome some of the challenges of developing an ideal hemophilia A gene therapy, such as durability and eligibility [132]. For example, they integrate into the target cell chromatin and are maintained during replication, which may be beneficial for long-term expression. In addition, the prevalence of pre-existing HIV immunity is low (an estimated 0.7% of adults aged 15-49 years have the disease) [132, 133], so this approach may benefit a broad population of patients. However, potential safety concerns exist, as lentiviruses may be associated with oncogenic potential, and manufacturing the required quality and quantity of lentiviruses for use in gene therapy is problematic [132]. Furthermore, long-term efficacy data in large animal models are lacking. Phase 1 studies using lentiviruses are ongoing or planned for YUVA-GT-F801 (Shenzhen Geno-Immune Medical Institute), ET3 (Expression Therapeutics) and Pleightlet (MUT6; Medical College of Wisconsin).

### 6.4 Gene editing/CRISPR

Gene editing to target the integration of a functional gene into the host genome, and thus improve disease phenotype, has generated considerable interest across many therapeutic areas. In hemophilia A, it has the potential to provide durable FVIII expression, but the nature and safety profile of the vehicle for intracellular delivery of the gene-editing machinery needs to be carefully considered. For example, if an AAV is used, then the problem of pre-existing immunity may still exist. In addition, off-target mutagenesis can occur [134], potentially leading to deleterious effects.

*In vitro* experiments and mouse models have shown that it is possible to use clustered regularly interspaced palindromic repeats (CRISPR)/Cas9 to express FVIII [135]. However, gene editing to treat hemophilia A is still in its infancy, and further research is required before it is clear how this technology will affect the treatment landscape.

## 7 CLINICAL IMPLICATIONS AND CONSIDERATIONS OF HEMOPHILIA A GENE THERAPY

Gene therapy can potentially be a transformational experience in clinical practice, with no need for regular FVIII infusions and improved quality of life. It may give patients the ability to live the life that they choose more of the time and have increased freedom [136]. In addition, gene therapy may reduce treatment burden and costs. In a US cost analysis model, hemophilia A gene therapy was predicted to be cost-saving compared to prophylaxis with clotting factor concentrates [137]. Furthermore, gene therapy could potentially resolve other treatment access issues [3].

However, several unknown risks within the patient journey remain, including the current lack of re-treatment options, should initial gene therapy fail to provide clinically relevant FVIII levels. In addition, the potential for any inhibitor development after gene therapy will need to be managed [3]. As discussed, individual variability in the FVIII expression level and the duration of the response are expected. Long-term data are needed to determine the average patient’s bleeding phenotype following the treatment with gene therapy, as well as a personalized assessment. It may be that some patients continue to require additional FVIII treatment to control acute bleeding events, particularly those associated with traumas, surgery, and/or intense physical activity, especially immediately after gene therapy. Furthermore, without very long-term data (*i.e*., decades), the potential oncogenicity of any gene therapy is an ongoing area for investigation and monitoring across all disease states. To address these concerns and others, there is a worldwide commitment to monitoring the long-term efficacy and safety of gene therapy, as well as collecting patient-reported outcomes (including *via* mobile application technology). The World Federation of Hemophilia Gene Therapy Registry has been conceived, in collaboration with a number of international bodies interested in hemophilia treatment, to serve as a global repository of lifelong data for patients with hemophilia who receive gene therapy [138].

Another consideration is that patients need to be aware of the constraints associated with participation in a hemophilia A gene therapy trial: regular visits, tests and observation in the first 52 weeks, long-term alcohol abstinence (6 months or longer), barrier contraception to avoid vector contamination of germline cells (usually up to 48 weeks post-infusion), short-term steroid treatment and long-term (multi-year) monitoring [138, 139]. Although no gene therapies for hemophilia A have been approved, the approach will hopefully move beyond clinical trials. At this stage, the choice of product will become an important decision for patients. Healthcare professionals will need to be prepared to guide them to pick the best individualized treatment option [140, 141].

## CONCLUSION

In conclusion, extensive preclinical research has supported the development of several gene therapies for hemophilia A. A number of AAV-based product candidates are currently in Phase 1–3 clinical trials, with the aim of assessing whether a clinically-relevant and sustained improvement of the disease phenotype is achievable while maintaining an acceptable safety profile. An ideal treatment solution is not currently available, and a personalized approach for each patient will be required. Every gene therapy is a combination of different design features, built on decades of research into genetics, AAV technology and capsid bioengineering.

Currently, several safety concerns regarding gene therapy remain, especially in terms of managing the immune response. Until the development of a re-dosing AAV option that circumvents immunogenicity or a long-lasting immune response, gene therapy remains a potentially once-in-a-lifetime opportunity. Furthermore, research is needed for patient subgroups previously excluded from trials, including children and people with comorbidities, such as active hepatitis and HIV. Future gene therapy patients should also be aware of lifestyle adjustments when choosing gene therapy (*e.g*., alcohol abstinence and other clinical trial requirements, including the commitment to long-term monitoring). For people with hemophilia to have greater freedom from burdensome treatment and potential long-term health complications, gene therapy is needed, and clinical study data will help answer key questions about the future of this new, potentially life-changing treatment modality.

## Figures and Tables

**Table 1:** Current hemophilia A gene therapy products that have entered clinical trials*

**Product / Study**	**Status at Time of Publication**
**AAV Vectors**	
**ASC618,** ASC Therapeutics	Phase 1/2 (NCT04676048)
**BAY 2599023,** Bayer/Ultragenyx Pharmaceutical	Phase 1/2 (NCT03588299)
**SPK 8016,** Spark Therapeutics/Roche	Phase 1/2 (NCT03734588)
**SPK 8011,** Spark Therapeutics/Roche	Phase 1/2 (NCT03003533)
**Giroctocogene Fitelparvovec,** Sangamo Therapeutics/Pfizer	Phase 3 (NCT04370054)
**Valoctocogene Roxaparvovec** (Roctavian^®^), BioMarin Pharmaceutical	Phase 3 (NCT03370913) Recommended for European conditional marketing authorization by the Committee for Medicinal Products for Human Use; awaiting final decision by the European Commission
**Lentiviral Vectors**	
**Auto CD34+PBSC,** Medical College of Wisconsin	Phase 1 (NCT03818763)
**YUVA-GT-F801,** Shenzhen Geno-Immune Medical Institute	Phase 1 (NCT03217032)
**CD68-ET3,** Expression Therapeutics	Phase 1 (NCT04418414)

*At the time of publication, no CRISPR/Cas or gene editing technologies for hemophilia A had entered clinical trials.

## LIST OF ABBREVIATIONS

EHL: Extended Half-Life
FVIII: Factor VIII
HCB: Hepatic Combinatorial Bundle
ITR: Inverted Terminal Repeat
SHL: Standard Half-Life
